# How do psychological treatments work? Investigating mediators of change

**DOI:** 10.1016/j.brat.2008.10.001

**Published:** 2009-01

**Authors:** Rebecca Murphy, Zafra Cooper, Steven D. Hollon, Christopher G. Fairburn

**Affiliations:** a Department of Psychiatry, Oxford University, Warneford Hospital, Oxford OX3 7JX, UK; b Department of Psychology, Vanderbilt University, Nashville, TN 37240, USA

**Keywords:** Treatment, Mediation, Mechanisms, Interpersonal psychotherapy, Cognitive behaviour therapy, Eating disorders

## Abstract

Little is known about how psychological treatments work. Research on treatment-induced mediators of change may be of help in identifying potential causal mechanisms through which they operate. Outcome-focused randomised controlled trials provide an excellent opportunity for such work. However, certain conceptual and practical difficulties arise when studying psychological treatments, most especially deciding how best to conceptualise the treatment concerned and how to accommodate the fact that most psychological treatments are implemented flexibly. In this paper, these difficulties are discussed, and strategies and procedures for overcoming them are described.

## Introduction

While a large body of research has established the efficacy and effectiveness of a range of psychological treatments, little is known about how they work. This is an important shortcoming as most are limited in their efficacy. To help make them more potent, it would be of great value to understand how they work as research could then focus on enhancing the effective elements whilst discarding those elements found to be redundant (Kazdin & Nock, 2003). As matters stand markedly differing views are held regarding how psychological treatments work. For example, some claim that they work exclusively through common, or “non-specific”, mechanisms (see Luborsky et al., 2002) a position that is hard to reconcile with the many studies that have identified treatment-specific effects (e.g., Clark et al., 2006; Fairburn, Jones, Peveler, Hope, & O'Connor, 1993). Opinions differ even when it comes to considering how a single psychological treatment works: for example, it has been argued that cognitive therapy for depression works by changing the content and structure of cognitive schema (Beck, Rush, Shaw, & Emery, 1979), through teaching compensatory skills (Barber & DeRubeis, 1989), or by establishing a metacognitive stance (Teasdale et al., 2002). To clarify such matters the processes responsible for treatment-induced change need to be identified and one step in this regard is the identification of mediators of change. What impedes such work, however, is the difficulty in designing and conducting mediational studies of psychological treatments. It is these difficulties, and the means of addressing them, that are the subject of this paper.

First, some concepts and terms need to be introduced. Mediators of treatment effects are variables which account for, in a statistical sense, at least some of the effects of treatment on the patient's outcome (Baron & Kenny, 1986). If the effects of treatment are mediated by a variable, this finding is consistent with the hypothesis that the treatment works by modifying this variable (the mediator). However, it must be stressed that mediation analysis involves merely identifying associations between putative variables: it does not establish that the identified relationship is causal in nature. The identification of mediators is, therefore, an initial step in establishing how treatments work, the next step being the testing of the causal status of any identified mediators by manipulating them. Thus, the value of identifying mediators lies in the narrowing down of the search for causal mechanisms.

Historically, there has been confusion and inconsistency over the use of the terms mediation and moderation. As defined by Kraemer, Wilson, Fairburn, and Agras (2002), moderators precede treatment, are uncorrelated with treatment and “explain”, in a statistical sense, individual differences in the effects of treatment. They indicate in whom and under what circumstances treatment has the most effect. In contrast, mediators are a consequence of treatment and “explain” in a statistical sense some of the effects of treatment on outcome. Thus, mediators correlate with treatment, are modified during treatment and this change precedes the effects of treatment on outcome.

## Investigating mediators in the context of randomised controlled trials

Randomised controlled trials provide an often-missed opportunity to investigate the mediators of treatment effects and guidelines have been proposed for doing this (Kazdin, 2007; Kraemer et al., 2002). The key points are as follows. First, the decision to perform a mediator analysis needs to be taken in advance as it will influence the choice of measures used and when they are applied. Next, hypotheses need to be formulated concerning the likely mechanisms of action of the treatment under study. The hypotheses are likely to be derived from the theory underpinning the treatment and the findings of prior research. Then, the treatment, the putative mediators and the outcome need to be operationalised and a suitable assessment protocol devised. The ideal situation is when mediators are investigated in the context of trials that include a comparison treatment or control condition as this can help to rule out the possibility that what appears to mediate change is simply a general effect of receiving treatment (e.g., an expectancy effect) or a naturally occurring change (e.g., regression to the mean) rather than the specific effect of the treatment under consideration. The nature of the comparison treatment is also relevant. Minimal comparison treatments that are less effective than the index treatment can serve as controls for the processes just described, whereas treatments that are equally effective but operate via different mechanisms – as illustrated below – may be used to ensure that change in the putative mediator is not a consequence of change in the outcome variable (“reverse causality”). Note that if more than one treatment is being compared, it is possible to study the mediators of action of both treatments simultaneously, again as illustrated below.

The timing of the measurements is of critical importance as change in the mediator needs to be shown to have occurred prior to change in the outcome of interest as otherwise it could merely be a secondary effect. Establishing temporal precedence is particularly challenging when the effects of the mediator are likely to be rapid as is often the case. One solution is to measure both the putative mediator and the outcome variable at frequent intervals throughout the period over which change is likely to occur. Simultaneous measurement of both the mediator and the outcome variable also allows one to control for the level of the outcome when predicting its subsequent change. It is important to stress that it is not sufficient to simply measure the level of the outcome variable at some later point in time, for example, at the end of treatment. This is because treatment may produce change in the outcome prior to the point at which the mediator is assessed with the consequence that some portion of the change observed in the mediator could be the result of a causal path from early change in the outcome variable to subsequent change in the mediator (DeRubeis et al., 1990).

With regard to statistical analysis, a variety of data analytic strategies and procedures have been developed to assess whether a putative mediator meets statistical criteria for mediation. Readers are referred to the following sources (among others): Baron and Kenny (1986); MacKinnon, Lockwood, Hoffman, West, and Sheets (2002); Shrout and Bolger (2002) and MacKinnon, Fairchild, and Fritz (2007).

## Studying the mediators of action of psychological treatments

Two particular challenges arise when studying the mediators of action of psychological treatments. First, psychological treatments differ in how they may be conceptualised. Some tend to be viewed as a complete unit rather than as a collection of procedures, whereas in others the opposite is the case. Interpersonal psychotherapy (IPT; Klerman, Weissman, Rounsaville, & Chevron, 1984; Weissman, Markowitz, & Klerman, 2000), for example, is usually conceptualised as a complete treatment or entity, as will be discussed below. In contrast, many cognitive behavioural treatments (CBT) comprise a variety of different procedural elements that may be viewed either in isolation or as an integrated group of techniques directed at a specific goal. For example, CBT for depression may be thought of either as a unit or as a treatment that comprises a number of distinct procedures including behavioural activation and cognitive restructuring (Jacobson et al., 1996). This distinction is important since it provides the possibility of either investigating the action of the treatment as a whole or that of its component parts.

The second matter is practical rather than conceptual. It is that all but the simplest psychological treatments are implemented in a flexible manner. In other words, their precise use is tailored to the individual patient's psychopathology, circumstances and progress at making change, and as a result the treatment is not identical in every case. For example, in most forms of CBT the precise time when particular procedures are implemented differs from patient to patient. Indeed, in some cases certain procedures may not be used at all. This flexibility, inherent to good clinical practice, substantially complicates research on mediation.

## Investigating the mediators of action of CBT and IPT in the treatment of eating disorders

There follows a description of the strategies and procedures that we have developed to address certain of the difficulties specified above. The context is the treatment of patients with eating disorders but the principles apply across different disorders and different treatments. In this particular case, two treatments are being studied, interpersonal psychotherapy for eating disorders (Fairburn, 1997; Murphy, Straebler, Cooper, & Fairburn, in press) and “enhanced” transdiagnostic CBT for eating disorders (CBT-E; Fairburn, 2008a; Fairburn et al., in press). Both treatments are outpatient-based and involve 20 treatment sessions over 20 weeks. Initially the sessions are twice weekly, then after 4 weeks they are weekly for 10 weeks, and finally there are three sessions 2 weeks apart. Both treatments are the sole interventions that the patients receive other than continuation antidepressant medication in some cases.

IPT and CBT-E differ markedly in their rationale, strategies and procedures, and are very likely to have different modes of action. IPT is designed to help patients overcome their eating disorder indirectly by resolving current problems in their interpersonal life. The treatment is derived from IPT for depression (Klerman et al., 1984; Weissman et al., 2000) and closely resembles it. It has three phases. The first generally occupies 3–4 sessions. The aim is to describe the rationale and nature of the treatment and to identify jointly one or more current interpersonal problems that will thereafter become the focus of treatment. In the second phase, these problems are examined in detail with the therapist helping the patient first to characterise them and then identify possible means of addressing them. In the final phase, the focus shifts to the future, the goals being to ensure that any interpersonal changes made in treatment are maintained and to minimise the risk of relapse in the longer-term.

CBT-E is the latest version of the leading empirically supported treatment for eating disorders (Fairburn, 2008b). Originally, it was a treatment for bulimia nervosa but it is now transdiagnostic in nature; that is, it is designed to be suitable for all forms of eating disorder whatever the DSM-IV diagnosis. Unlike IPT, CBT-E comprises a collection of strategies and procedures focused directly on the characteristic psychopathology of eating disorders. While individual procedures target specific elements of this psychopathology (e.g., binge eating, body shape checking), the procedures are designed to be used in concert, the goal being to address all the main maintaining mechanisms operating in the individual patient. Thus, CBT-E can be conceptualised either as a collection of discrete procedures, each of which has its own mechanism of action, or as a complete unit. Complicating the investigation of CBT-E is the fact that it is designed to be used very flexibly with the choice of procedures and the timing of their implementation being tailored to the needs of the individual patient.

There follows a description of how we are attempting to identify the mediators of action of these two very different treatments starting with an outline of our hypotheses concerning how they might operate.

## Hypothesised mediators of change in IPT

Little has been written about how IPT works. Drawing both on the original theory underpinning IPT (Klerman et al., 1984) and on our experience observing how patients change during treatment, we have formulated four hypotheses concerning the mechanisms through which IPT might achieve its effects. It is important to note that we do not view these as the sole mechanisms through which IPT operates nor are the hypotheses mutually exclusive. For example, in bulimia nervosa there is a rapid initial change in the frequency of binge eating and vomiting that is much the same in IPT and CBT (Fairburn, Agras, Walsh, Wilson, & Stice, 2004; Wilson, Fairburn, Agras, Walsh, & Kraemer, 2002). This is likely to be the result of common “non-specific” processes associated with starting treatment and, interestingly, its magnitude is a powerful predictor of outcome (Agras, Crow et al., 2000; Fairburn et al., 2004). Nevertheless, we hypothesize that in addition to these early non-specific processes (which operate in both IPT and CBT), IPT operates through four IPT-specific mechanisms.

1 *The reduction in eating disorder features is largely mediated by a decrease in the severity of the interpersonal problem(s) targeted in treatment*. In this case, the putative mediator is a reduction in the severity of the specific targeted interpersonal problem(s).
2 *The reduction in eating disorder features is largely mediated by an increase in interpersonal self-efficacy with regard to the specific problem(s) targeted in treatment*. Thus, in this case the putative mediator is an increase in the strength of the patient's belief that he or she is capable of overcoming the targeted interpersonal problem(s).
3 & 4 *The reduction in eating disorder features is largely mediated by an increase in general interpersonal self-efficacy (hypothesis 3) or self-esteem (hypothesis 4)*. In the case of hypothesis 3, the proposed mediator is an increase in the strength of the patient's belief that he or she is capable of overcoming interpersonal difficulties in general rather than just those which have been the focus of treatment. In hypothesis 4, the putative mediator is an increase in self-esteem.

The nature of these four putative mediators is such that it is likely that they take considerable time to change. Therefore, in the case of all four hypotheses it is predicted that the critical change in the mediator takes place during the later stages of treatment and that the change in the eating disorder largely takes place subsequent to this. This prediction is consistent with the unexplained finding that patients with bulimia nervosa who receive IPT show significantly less change in their frequency of binge eating and vomiting than those who receive CBT, a difference that disappears over follow-up due to continuing improvement in the IPT patients and little or no change among those who received CBT (Agras, Walsh, Fairburn, Wilson, & Kraemer, 2000; Fairburn et al., 1993).

## Hypothesised mediators of change in CBT-E

In the case of CBT-E, we have chosen to focus on the mediators of action of four specific treatment procedures rather than that of the treatment as a whole. Each procedure addresses a specific psychopathological process thought to be central to the maintenance of most eating disorders, and each is described in detail in the full treatment guide (Fairburn, 2008a).

1 The “weekly weighing” procedure – *It is predicted that the reduction in weight concern which occurs early on in CBT-E is largely mediated by a reduction in the frequency of weight checking (as a result of the “weekly weighing” procedure)*. Patients with eating disorders are extremely concerned about their weight (i.e., the number on the scale). They often check their weight very frequently and as a result become focused on inconsequential weight changes (Fairburn, 2008b). This overconcern with weight, and the associated fear of weight gain, maintains strict dietary restraint and is a barrier to patients changing their way of eating. It is addressed early on in CBT-E by the weekly weighing procedure in which the therapist and patient jointly check the patient's weight once a week and then together interpret the resulting finding in the light of education and prior readings. Patients are helped not to weigh themselves between these times.
2 The “regular eating” procedure – *It is predicted that the reduction in the frequency of binge eating which occurs early on in CBT-E is largely mediated by the adoption of a pattern of regular eating (as a result of the “regular eating” procedure)*. Patients with eating disorders have a distinctive way of eating. Their temporal pattern of eating tends to be characterised by delayed eating and the avoidance of meals or snacks, or by a highly unstructured way of eating (Fairburn, 2008b). Regular eating, like weekly weighing, is introduced early on in treatment. It involves helping patients establish a daily pattern of eating characterised by three planned meals and two snacks with no eating in between. Regular eating of this type has a variety of effects one of which is to displace binge eating.
3 The “dietary rules” procedure – *The reduction in dietary restraint seen in the mid-to-late stages of CBT-E is largely mediated by erosion of the belief that adherence to strict dietary rules is necessary to prevent binge eating, weight gain or fatness (as a result of the “dietary rules” procedure)*. The great majority of patients with eating disorders engage in a highly distinctive form of dieting characterised by attempts to adhere to multiple strict and demanding dietary rules (Fairburn, 2008b). Doing so is valued by the patients. The “dietary rules” procedure is designed to address this form of dieting. It involves identifying patients' dietary rules and highlighting the fact that they substantially impair their day-to-day life and maintain their eating problem, and so need to be targeted in treatment. Patients' fears about breaking these rules are identified and challenged using behavioural experiments in combination with relevant education about the triggers of binge eating and the processes underlying weight gain and shape change. As a result, patients learn that the feared consequences of breaking these rules do not occur and consequently the rules become viewed as a problem rather than as a strength. This erosion of the belief that adherence to strict dietary rules is necessary to prevent binge eating, weight gain or fatness leads patients to moderate and eventually abandon their dietary restraint. The use and timing of this intervention is tailored to the needs of the individual patient based on an evaluation of the importance of “dietary rules” in maintaining whatever eating disorder psychopathology remains after the first stage of treatment.
4 The “body shape checking” procedure – *The reduction in concern about body shape which occurs in the mid-to-late stages of CBT-E is largely mediated by a decrease in body shape checking (as a result of the “body shape checking” procedure)*. Patients with eating disorders typically engage in frequent shape checking (using multiple methods including body pinching, body measuring, and scrutinising themselves in mirrors) (Fairburn, 2008b). In the “body shape checking” procedure, patients are first made aware of their body checking (much of which may occur outside their awareness) and its adverse effects. Patients are then helped to make strategic changes to their shape checking and to evaluate the significance of the resulting effects. This results in a decrease in their concerns about body shape. In common with the dietary rules procedure, the use and timing of this procedure is personalised to the individual patient based on the importance of shape concern in maintaining their remaining eating disorder psychopathology.

In contrast with IPT's hypothesised mechanisms of action, the four CBT procedures are thought to operate rapidly with there being a short time lag between change in the putative mediator and change in the relevant outcome variable.

## Research design

**Fig. 1:**
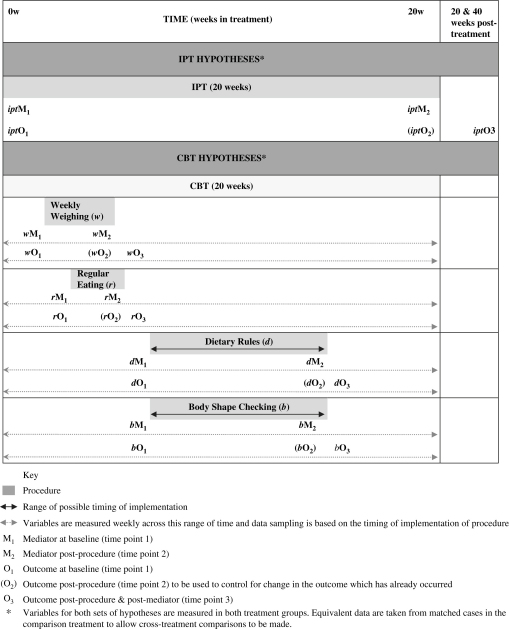
Schematic diagram illustrating the research design used to identify mediators of action of IPT and CBT-E in the treatment of patients with eating disorders.

In terms of research design, the investigation of the four putative mediators of action of IPT is relatively straightforward (and is illustrated in Fig. 1). The main points are as follows:

1 IPT is conceptualised as a unit;
2 the mediator is measured at baseline and at the end of IPT (i.e., 20 weeks later);
3 the outcome of interest (level of eating disorder psychopathology) is measured at the outset of IPT and 20 and 40 weeks after the end of treatment (i.e., well after the end-of-IPT measurement of change in the putative mediator, thereby addressing temporal precedence). The level of eating disorder psychopathology is reassessed at the end of treatment so that any incidental in-treatment change may be controlled for in the analysis;
4 equivalent data are taken from individually matched CBT-E cases to allow cross-treatment comparisons to be made.

The same principles are used to address the four CBT-E hypotheses. However, in this case there are two additional complexities. The first is that change in the mediator is thought to occur rapidly and as a result there may be a relatively short interval before change in the outcome occurs. The second is that the third and fourth of the CBT-E procedures under study are not introduced at set points in treatment. As a result, the design has to be modified in four ways (Fig. 1):

1 the timing of implementation of each of the four CBT-E procedures is recorded on a case-by-case basis so that it is known exactly when they took place;
2 each mediator and outcome variable is measured frequently and simultaneously throughout treatment;
3 the data needed for the mediational analyses are selected on a patient-by-patient basis depending on when each of the four procedures was implemented. In each case, the data on the level of the mediator is extracted just before the implementation of the procedure concerned, and also at the end of its implementation. Similarly, data on the level of the outcome variable is extracted just prior to the onset of the implementation of the procedure and shortly after the reassessment of the level of the mediator. Thus, in this way a “floating”, but predetermined, individualised dataset is extracted for analysis;
4 equivalent data are collected from an individually matched IPT case so that cross-treatment, temporally matched, comparisons may be made.

## Conclusions

In this paper, we have discussed in principle how research on mediators of change may be of help in identifying potential causal mechanisms through which psychological treatments operate. Following Kraemer et al. (2002), we have argued that such work can fruitfully be combined with outcome-focused randomised controlled trials. We have noted that certain conceptual and practical challenges arise when studying psychological treatments, two being particularly important. The first is how best to conceptualise the treatment under investigation, the core decision being whether to view it as a unit or as a collection of procedures each with its own mode of action. The second stems from the fact that most psychological treatments are implemented flexibly. We have discussed potential ways of addressing these problems based on our work evaluating the mediators of action of IPT and CBT-E in the treatment of patients with eating disorders. The same principles should apply to the investigation of other psychological treatments and other psychiatric disorders.
